# Pain in dementia: prevalence and associated factors: protocol of a multidisciplinary study

**DOI:** 10.1186/s12877-015-0025-0

**Published:** 2015-03-21

**Authors:** Janine van Kooten, Suzanne Delwel, Tarik T Binnekade, Martin Smalbrugge, Johannes C van der Wouden, Roberto SGM Perez, Didi Rhebergen, Wouter WA Zuurmond, Max L Stek, Frank Lobbezoo, Cees MPM Hertogh, Erik JA Scherder

**Affiliations:** Department of General Practice and Elderly Care Medicine and the EMGO+ Institute for Health and Care Research, VU University Medical Center, Van der Boechorststraat 7, 1081 BT Amsterdam, The Netherlands; Department of Clinical Neuropsychology VU University, Amsterdam, The Netherlands; Department of Oral Kinesiology, Academic Centre for Dentistry Amsterdam (ACTA), University of Amsterdam and VU University Amsterdam, MOVE Research Institute Amsterdam, Amsterdam, The Netherlands; GGZ InGeest /Department of Psychiatry and the EMGO+ Institute for Health and Care Research, VU University Medical Center, Amsterdam, The Netherlands; Department of Anesthesiology, VU University Medical Center, Amsterdam, The Netherlands

**Keywords:** Aged, Alzheimer’s disease, Frontotemporal dementia, Vascular dementia, Dementia with Lewy Bodies, Pain, Orofacial pain, Autonomic responses, Neuropsychiatric symptoms, Quality of life

## Abstract

**Background:**

Pain is a common problem in people with dementia, however the exact prevalence of pain in dementia subtypes, e.g. Alzheimer’s Disease (AD), Vascular Dementia (VaD), Frontotemporal Dementia (FTD) and dementia with Lewy Bodies (DLB), is unknown, as is the relation between pain and the different subtypes of dementia. In this study, the prevalence of pain in people with dementia will be investigated per dementia subtype and the relationship between the various subtypes of dementia and the presence of specific types of pain (i.e. musculoskeletal pain, neuropathic pain and orofacial pain) will be examined. Secondly, associations between various types of pain, cognitive functioning, neuropsychiatric symptoms and quality of life in people with dementia will be examined. A third purpose is to study the value of the assessment of autonomic responses in assessing pain in people with dementia. Finally, the effect of feedback to the attending physician on the presence of pain, based on examination by investigators with backgrounds in neuropsychology, geriatric dentistry and elderly care medicine, will be evaluated.

**Methods/Design:**

A cross-sectional, partially longitudinal observational study in 400 participants with dementia, aged 60 years and older. Participants will be recruited from an outpatient memory clinic and dementia special care units. All participants will be examined by an elderly care medicine trainee, a dentist with experience in geriatric dentistry, and a neuropsychologist. The primary outcome is presence of pain. Secondary outcomes will include oral health, autonomic responses to pain stimulus, vital sensibility and gnostic sensibility, musculoskeletal examination, cognitive functioning, neuropsychiatric symptoms, and quality of life.

**Discussion:**

This study will help to enhance our knowledge regarding the prevalence of different types of pain in different dementia subtypes i.e. AD, VaD, FTD and DLB. This study also aims to contribute to a better understanding of oral health status in people with dementia, the use of autonomic responses in the assessment of pain in people with dementia and the relationships between pain and cognitive symptoms, neuropsychiatric symptoms and quality of life in people with various dementia subtypes and in different stages of the disease.

## Background

Pain is a common problem in people with dementia. The prevalence of pain in people with dementia is high [1-4]; there is good agreement in both large and small studies that about 50% of the people with dementia regularly experience pain [5]. This is not surprising, considering that advanced age is an important risk factor for developing dementia, as well as for pain [6,7].

Pain in dementia is associated with occurrence of neuropsychiatric symptoms [8,9], and also with a decline in cognitive functioning, as well as in performance of Activities of Daily Living (ADL) [10]. Next to neuropsychiatric symptoms, pain is the most cited reason for a decrease in quality of life in dementia [11]. Therefore, recognition and adequate treatment of pain in people with dementia should have high priority.

Although knowledge about the differences in pain prevalence between dementia subtypes is scarce, it is suggested that differences in neuropathology between dementia subtypes could be of vital importance in the treatment of pain [12]. Results from previous small studies showed that specific neuropathological changes may affect pain experience and pain perception differently in the four commonest dementia subtypes [12], i.e., Alzheimer’s Disease (AD), Vascular Dementia (VaD), Dementia with Lewy Bodies (DLB), and frontotemporal dementia (FTD). It is suggested that with a preponderance of white matter lesions, pain experience in VaD may increase compared to other dementia subtypes [12], while in AD and FTD it is hypothesized that neuropathological changes like gray matter atrophy lead to an increase in pain tolerance [13-15].

Current knowledge about the prevalence of specific types of pain, e.g., musculoskeletal pain or neuropathic pain, and causes of pain in people with dementia is limited. Nociceptive pain of musculoskeletal origin is considered the most prevalent pain type, but orofacial pain and neuropathic pain are also likely to have considerable prevalence rates [16,17]. For example, orofacial pain is one of the most prevalent types of pain in older people, and has hardly been studied in people with dementia [17,18]. Assessment of the specific type of pain and classifying its probable causes would be useful as a complement for the assessment of pain in people with dementia, and would provide a sound basis for appropriate pain treatment when combined with dementia subtyping in people with dementia.

An important problem encountered in the assessment of pain in patients with dementia, especially in more advanced stages, is that assessment often is based on observation and interpretation of behavior, for which the validity is unequivocally established [5,19,20]. More objective methods for assessing pain in a more indirect way in this population are therefore warranted. Assessing autonomic responses to pain might be such a method [16,21].

Considering the limited number of studies on pain in each subtype of dementia, and the absence of studies on type of pain in dementia subtypes and in different stages of dementia, we developed a unique multidisciplinary approach. Investigators with backgrounds in neuropsychology, geriatric dentistry, elderly care medicine, and pain medicine will study in close cooperation the presence and type of pain, in various stages of the four main subtypes of dementia (AD, VaD, FTD and DLB). In a subset of patients we will explore the feasibility of measuring autonomic responses as a tool to assess the presence of pain.

### Research aims

The primary aim of this study is to investigate the prevalence and intensity of pain (subtypes) in the four main dementia subtypes and to examine the relationship between the different types of pain and the different dementia subtypes, i.e., AD, VaD, FTD and DLB.

Secondary aims are:

- to examine the associations between neuropsychiatric symptoms and pain, quality of life and pain, and cognitive function and pain for all different dementia subtypes;
- to explore whether autonomic responses can be used for assessing pain in patients with more advanced stages of dementia;
- to test the psychometric properties of the newly developed Orofacial Pain Scale for Non Verbal Individuals (OPS-NVI).
- to examine the oral health of older adults with dementia.
- evaluate the effect of providing feedback on the presence of pain (including treatment advice), to the attending physician and/or dentist.

## Methods/Design

### Study design

This protocol describes an observational, cross-sectional and partly longitudinal cohort study. Measurements take place at baseline, and for the people with pain at baseline, a follow-up pain measurement takes place after 3 months.

### Recruitment

To ensure this study population covers the broad range of the elderly population with dementia, participants will be recruited in different stages of dementia. Participants with mild to moderate dementia, will be recruited at the outpatient memory clinic of the Center of Geriatric Medicine Amsterdam (COGA), VU University medical center. Participants with moderate to severe dementia will be recruited from Amstelring, a health organization delivering specialized care to residents with dementia in 17 nursing homes covering a large area in the northwestern part of the Netherlands, including Amsterdam.

### Inclusion criteria

Participants who meet the following criteria will be included: aged 60 or older, diagnosis of dementia, i.e. AD, VaD, FTD, or DLB, and a signed informed consent by the participant or legal representative.

### Exclusion criteria

Primarily mentally disabled persons who subsequently develop dementia, e.g. people with Down’s syndrome and AD, will be excluded. Persons who indicate either verbally or non-verbally, that they do not wish to participate will be excluded as well, despite of given consent by a legal representative.

### Ethical approval and informed consent

The study protocol has been approved by an accredited Medical Ethics Review Committee (VU University Medical Center; NL.43861.029.13).

Informed consent will be obtained of all participants. For participants unable to give consent, the legal representative will be asked to sign an informed consent form.

### Measurement instruments

Variables measured in this study (primary outcome measures, secondary outcome measures, demographic, and control variables) are summarized in Table 1. These will be assessed by using a combination of self-report instruments and observational instruments, which are completed by caretaker or by a contact nurse.

**Table 1:** **Measurement instruments**

**Instrument**	**Source**	**Time (in minutes)**	**T0 memory clinic**	**T0 nursing home**	**T1 nursing home**
Rey Auditory Verbal Learning Test (15 Word Test)	1	15	*		
Amsterdam Dementia Screening Test -Meander (ADS-6)	1	5	*		
Behavioural Assessment of the Dysexecutive Syndrome (BADS) – Rule Shift Cards	1	5-10	*		
Brief Pain Inventory (BPI)	1	5	*	*	*
Dementia Quality of Life (DQoL)	1,2	5	*		
Faces Pain Scale – Revised (FPS-R)	1	3	*	*	*
Global Deterioration Scale (GDS)	3	1		*	
Katz-ADL					
Mini Mental State Exam (MMSE)	1	10	*	*	
Mobilization Observation Behaviour Intensity Dementia-2 (MOBID-2) Pain Scale	3	5		*	*
Neuropsychiatric Inventory- Nursing Home (NPI-NH)	3	15		*	
Neuropsychiatric Inventory- Questionnaire (NPI-Q)	2	5	*		
Numeric Rating Scale (NRS)	1	2	*	*	*
Orofacial Pain Scale for Non Verbal Individuals (OPS-NVI)	3	10	*	*	*
Oral Examination	1	20	*	*	
Pain Assessment IN Advanced Dementia (PAINAD)	3				
Quantitative Sensory Testing (QST)	1	15	*	*	
Qualidem	3	10		*	
Revised Index for Social Engagement (RISE)	3	5		*	
Rey Complex Figure Test (RCFT)	1	5-10	*		
Stroop Color-Word Test	1	5	*		
Structured physical examination	1,3	15	*	*	
Trail Making Test (TMT)	1	5	*		
Verbal Descriptor Scale (VDS)	1	2	*	*	*
Verbal Fluency Test (VFT) - Animals	1	3-10	*		
Visual Association Test (VAT)	1		*		
VU University- Ambulatory Monitoring System (VU-AMS)	1	10	*	*	

1 = Patient, 2 = Caretaker, 3 = Health professionals, T0 = baseline, T1 = after 3 months.

#### Primary outcomes

The primary outcome will be: the presence and intensity of pain in dementia subtypes. Pain will be assessed, using a combination of methods concurrently.

In *participants who are able to communicate about the presence of pain*, the Brief Pain Inventory (BPI) will be applied [22]. The BPI is a questionnaire, assessing the severity of pain by four numeric rating scale items and, explores the degree to which pain interferes with daily activities by seven interference items. The BPI is a valid and reliable assessment tool for pain assessment in epidemiological studies [22]. Concurrently with the BPI, the participant will be asked to score the intensity of pain, when he or she is in pain, on a self-report scale. The Numeric Rating Scale (NRS), Verbal Descriptor Scale (VDS) [23-25], and Faces Pain Scale Revised (FPS-R) [26,27] can be used for this purpose, dependent on understanding by the participant of each of the scales.

In *participants with difficulties to communicate their pain*, a contact nurse will be asked to complete the Pain Assessment IN Advanced Dementia Scale (PAINAD) [28]. The PAINAD is a brief and easy to administer observational scale, especially used in people with advanced dementia. The reliability and validity of the PAINAD have been studied extensively and a cut-off score of 2 has been recommended to indicate the probable presence of pain [28-32].

Apart from these pain assessment tools, the Mobilization Observation Behaviour Intensity Dementia-2 (MOBID-2) Pain Scale [33] will be administered in all participants by a contact nurse to measure pain, using standardized movements. The MOBID-2 is a reliable, valid and time effective instrument to measure pain in people with advanced dementia [33]. For this study, the MOBID-2 was translated into Dutch, based on the guidelines for cross-cultural adaptation [34]. The translated version showed good feasibility in a pilot study. An overall score of NRS ≥ 3 in the MOBID-2 will be regarded as clinically relevant pain [33].

For the measurement of orofacial pain, Delwel and Lobbezoo developed the Orofacial Pain Scale for Non Verbal Individuals (OPS-NVI), based on [17] and [35], which demonstrated good face validity and usability in a pilot study. For further validation of the OPS-NVI, a number of steps will be performed. First, the verbal and nonverbal participants will be observed using the OPS-NVI, during rest, drinking, eating, and mouth care. These activities will be observed for indicators of pain behavior and the pain intensity will be assessed by two observers, whenever possible. Second, the group of verbal participants will be asked about the presence of pain and, if pain is present, the pain intensity with the NRS, VDS, and FPS-R. Third, a standardized dental examination will be performed in both verbal and nonverbal participants, to evaluate their oral health, which will be described hereafter. The results of observation, self-assessment and the dental examination will be compared in order to validate the OPS-NVI.

#### Secondary outcomes

Secondary outcomes include oral health, autonomic responses to a pain stimulus, vital sensibility, musculoskeletal examination, cognitive functioning, neuropsychiatric symptoms, and quality of life*.*

#### Oral health

Oral health will be evaluated by a dentist with experience in geriatric dentistry, performing a structured oral examination, including history taking, extraoral, functional, intraoral, and (whenever indicated) additional examination.

The function of the temporomandibular joint will be assessed with an Active Mandibular Examination (AME). The participant will be asked to make different jaw movements and the dentist will measure the excursions [36]. The presence or absence of pain will be observed; if the procedure is too painful, it will be stopped [37]. Whenever applicable, removable prosthetics will be examined for hygiene, retention, and occlusion/articulation [38]. The status of the oral tissues will be determined by examining the lips, cheeks, palate, tongue, floor of the mouth, alveolar ridges, saliva, and mouth odor. These elements will be classified as healthy or abnormal by the dentist [38]. The number of opposing molars, the occlusal units (OU), will be counted as an indication of the chewing ability [39]. The status of the participant’s natural teeth will be scored with the Decayed Missing and Filled Teeth (DMFT) Index [40]. For this index, the number of teeth with caries, missing teeth, and teeth with fillings will be recorded. Also the amount of dental wear will be quantified and itemized [41]. The periodontal status will be determined by measuring the periodontal pockets according to the Dutch Periodontal Screening Index (DPSI) [42] and the physiological mobility of the teeth [43]. The oral hygiene will be measured, according to the Plaque Index by Silness and Loe [44]. Somatosensory changes of the face will be examined with brief quantitative sensory tests for orofacial pain. Three simple tests will be used: a cotton swab, pinprick, and brush test. The three innervation areas of the N. Trigeminus will be tested: above the eyebrow, under the eye and at the lower lip. The results will be used to determine if a participant has signs of neuropathic pain in the orofacial region [45]. For determining of signs of neuropathic pain elsewhere in the body, testing of vital and Quantitative Sensory Testing (QST) will be done (see below).

The oral examination will be evaluated and given as feedback to the participant, caretakers and/or medical staff.

### Autonomic responses to pain stimulus

Autonomic responses to pain will be measured with the VU University Ambulatory Monitoring System (VU-AMS), a certified device that records the electrocardiogram (ECG) and changes in thorax impedance (dZ) to determine Inter-Beat-Interval (IBI), Pre Ejection Period (PEP), and Respiratory Sinus Arrhythmia (RSA). In addition, Skin Conductance Level (SCL) will be recorded using the VU-AMS by two electrodes (Biopac TSD203) combined with their isotonic electrode gel (GEL101) on the index and middle finger [46-48].

At the outpatient memory clinic autonomic responses to a pain stimulus in the form of a venipuncture will be measured in all patients. First the patients will be asked to rate their current pain on the NRS followed by a two minute rest and a baseline measurement. Next, a venipuncture will be performed by a geriatric nurse and directly after this procedure the patient will be asked to rate the pain intensity of the venipuncture on the NRS. Subsequently, a second baseline measurement will be performed during a three-minute rest period. The patients will be asked to rate their current pain after the second baseline measurement to ensure that their pain level is not increased by sitting still for a prolonged period of time.

In nursing home residents, autonomic responses to pain stimuli are only measured in patients who undergo changes of wound dressings as part of usual care in the treatment of pressure ulcers. First the patients who are able to communicate, will be asked to rate their current pain level on the NRS followed by a five minute baseline measurement in rest. After the baseline measurement, the wound treatment procedure will be explained. The renewal of the wound dressing will be utilized as a painful episode. The current wound dressing will be removed, the wound cleaned and new dressing will be applied. Afterwards, the pain intensity of the treatment episode will be assessed in verbal patients by using the NRS. Finally, another 5 minute baseline measurement in rest will be performed.

#### Vital sensibility

Vital sensibility will be assessed by Quantitative Sensory Testing (QST), including touch perception, sharp/dull distinction and temperature discrimination. All sensory procedures will be administered to the inside of both lower arms. This site is often used in somatosensory studies [49,50] because of the high density of intra-epidermal nerve fibers [51]. QST will only be performed in patients with a Mini Mental State Examination (MMSE)-score ≥ 14.

The touch perception threshold will be assessed by nylon monofilaments based on the Von Frey hairs (Somedic AB, Sweden). A maximum number of 19 filaments will be administered to the inside of both lower arms, until the participant indicates the sensation of touch. False positive responses will be controlled by administering a null stimulus (no filament administered). The monofilaments increase in diameter, resulting in an increase in force that is applied to the skin. The monofilaments are numbered in ascending order. The number of the first monofilament resulting in a response will be used as an outcome.

The ability to sense the difference between sharp and dull stimuli will be assessed using the Neuropen (Owen Mumford, U.S.A.). This instrument contains both a monofilament for touch and a tip for sharpness. The monofilament will be pressed to the skin surface at a 90° angle until it bows (10 g) and subsequently hold in this position for 2 seconds. The sharp tip will be pressed to the skin surface at a 90° angle, until the marker is within the 40 g marker zone and subsequently held in this position for 2 seconds. Number of errors (maximum of 6) will be used as the outcome measure.

For temperature discrimination the Rolltemp (Somedic AB, Sweden) will be applied in random order to the left forearm (total of 3 times) and to the right forearm (total of 3 times). The subject will have to indicate whether the stimulus was cold or warm. The rollers will be placed in their docking stations between trials so their temperature is either 25 °C or 40 °C. Compared with the normal human skin temperature of 32 °C, the rollers will be perceived as either cold or warm, by humans with normal temperature sensitivity. The number of errors (maximum six) will be used as an outcome measure.

#### Musculoskeletal examination

A structured musculoskeletal examination will be performed by a physician in patients with clinically pain to determine whether the pain originates from the musculoskeletal system. Clinically relevant pain is either defined as BPI score ≥ 3, or self-report score ≥ 3, or MOBID-2 pain score ≥ 3, or PAINAD score ≥2.

#### Cognitive functioning

Cognitive functioning will be measured using the following memory measures: (1) the 15 Word test [52,53] adapted from the Rey Auditory Verbal Learning Test [54], (2) the Meander from the Amsterdam Dementia Screening Test [55], (3) the Rule Shift Cards Tests subtest of the Behavioural Assessment of the Dysexecutive Syndrome [56], (4) the Rey Complex Figure Test [57], (5) the Stroop Color-Word Test [58], (6) the Trail Making Test [59], (7) the Verbal Fluency Test Animals [60], (8) the Visual Association Test [61], and (9) the Mini Mental State Examination (MMSE) [62], a brief instrument used to screen for cognitive impairment and well validated in both research and clinical practice [63,64]. In nursing home patients only the MMSE will be used for measuring cognitive functioning.

#### Neuropsychiatric symptoms

Neuropsychiatric symptoms will be measured with a brief questionnaire form of the Neuropsychiatric Inventory (NPI-Q) [65] in participants at the outpatient memory clinic, and with the Neuropsychiatric Inventory Nursing Home Version (NPI-NH) [66] in nursing home residents. The NPI-Q is a revised version of the original NPI and has been validated against the original NPI [67]. The NPI-NH is developed for rating by professional caregivers within institutions and proved to be valid and reliable for trained nursing staff [66,68]. The NPI-NH is the only nursing home instrument for a broad range of neuropsychiatric symptoms that has been translated into Dutch [69].

#### Quality of life

Quality of life will be assessed by the Dementia Quality of Life instrument (DQol) [70] or the Qualidem [71,72]. The DQol is a valid instrument to assess the quality of life in persons with a mild to moderate stage of dementia. In people with moderate to severe dementia, the Qualidem will be used, which is a reliable and valid instrument that provides a quality of life profile of persons living in nursing homes [71,72].

In addition to quality of life, social participation will be measured using the Revised Index for Social Engagement (RISE) [73]. The RISE is a valid instrument for assessing social engagement in nursing home residents and contains six questions about the social interaction of the participant. A contact nurse who is familiar with the participant completes this questionnaire [73].

#### Dementia subtype and Stage

Participants recruited at the outpatient memory clinic will be screened for dementia, including Computed Tomography (CT) or Magnetic Resonance Imaging (MRI). The clinical diagnosis will be established by consensus within a multidisciplinary consultation. The National Institute of Neurological and Communicative Disorders and Stroke (NINCDS) and the Alzheimer’s Disease and Related Disorders Association (ADRDA) criteria for Alzheimer dementia [74], the National Institute of Neurological Disorders and Stroke Association (NINDS) Internationale pour la Recherche et l’Enseignement en Neurosciences (AIREN) criteria for vascular dementia [75], the revised criteria for FTD [76], and the revised criteria for DLB [77] are used. Participants recruited from dementia special care units will have a diagnosis of dementia usually based on DSM-IV criteria (2000), and the medical record will be reviewed for a probable subtype diagnosis determined by a geriatrician, neurologist, elderly care physician, or old age psychiatrist, and preferably completed by CT or MRI.

Dementia stage will be determined using the Global Deterioration Scale (GDS), a well-validated seven point scale [78]. The GDS defines seven stages of dementia, ranging from ‘no global impairment’ (1) to ‘very severe global impairment’ (7) [78].

#### Demographics, baseline, and control variables

Patient characteristics (i.e. sex and age) will be derived from medical records. The Charlson co-morbidity Index [79] will be used to classify co-morbidities, while ADL performance will be measured by the Katz-ADL [80]. The Katz-ADL assesses functional status and is used extensively in the field of elderly care medicine and geriatrics.

#### Medication

Information on medication use will be derived from the medical records at the outpatient memory clinic. To ensure a complete overview of pain medication, participants will explicitly be asked about their use of over-the-counter pain medication. In the nursing homes information on medication use will be derived from the pharmacists’ electronic patient records.

### Study procedure

#### Study measures after consent at the outpatient memory clinic

Measurements will be performed during the memory-screening day in the outpatient clinic. Since several measurements are already part of the standard routine, for research purposes the following measurements will be added, i.e. a structured pain assessment, an oral examination, the measurements of autonomic responses and sensibility (touch perception, sharp/dull distinction, temperature discrimination), the presence of neuropsychiatric symptoms, and the measurement of quality of life. Demographics, dementia subtype diagnosis, cognitive performance (i.e. neuropsychological assessment), ADL (i.e. Katz-ADL), co-morbidity (i.e. Charlson co-morbidity Index), and prescribed medication will be obtained from medical notes.

At the start of the memory-screening day, the neuropsychologist will install the VU-AMS device after which the VU-AMS protocol will be executed.

Participants will undergo a physical examination by a physician from the outpatient clinic; for this study, the physical examination will comprise a structured examination of the musculoskeletal system. Besides, patients will undergo QST by a trained neuropsychologist or research assistant. A dental examination will be performed by a dentist with experience in geriatric dentistry.

If pain is observed in participants, recruited in the outpatient memory clinic, feedback about the pain and the most probable cause(s) will be given to the attending physician.

#### Study measures after consent in nursing homes

To reduce participant burden, measurements in nursing home residents will be split into three parts, which will preferably be performed on 3 different days; i.e. (1) structured pain assessment including musculoskeletal examination when clinically relevant pain is present, (2) oral examination, and (3) cognitive assessment, QST and VU-AMS, if applicable. Structured pain assessment includes the BPI, self-report-scales, or PAINAD. Besides, all participants will be observed for presence of pain during morning care using the MOBID-2. Trained research assistants will collect data on neuropsychiatric symptoms, quality of life, participation, and ADL by interviewing the contact nurse. Dental examination will be performed by a dentist. Cognitive assessment will be done by a neuropsychologist. QST will only be assessed in patients with a MMSE-score ≥ 14, and the VU-AMS will only be used in nursing home residents who undergo changes of wound dressings as part of usual decubitus care.

If pain is observed in nursing home residents during baseline measurement, feedback about the type(s) of pain and a differential diagnosis of the most probable cause(s), including information about treatment options, will be given to the attending physician. Reassessment of these participants will take place after three months with the same pain measurement instruments as applied at baseline. During this reassessment, information about pain treatments in the last three months will be collected to evaluate the effect of feedback to the attending physician.

### Sample size and power analysis

Our first objective is to assess the prevalence of pain in dementia subtypes. To date, no clear estimates of the prevalence of pain in dementia subtypes are known. Therefore, we used an assumed prevalence of 50% for our sample size calculation. Aiming at a precision of 5 percentage points (i.e., estimate +/− 2.5%) with a significance level (alpha) of 0.05 and a power (beta) of 0.95, we will need to recruit 384 participants. Taking into consideration that prevalence measurements do not require follow-up, we did not correct for attrition.

To analyze the assumed association between neuropsychiatric symptoms and pain, quality of life and pain, and cognitive functioning and pain in all different dementia subtypes, multivariable linear regression analyses will be used. Power depends on the amount of explanatory variables and therefore we decided to include a maximum of 10 explanatory variables in our analyses. Based on the sample size calculations, using the software-tool ‘G*power version 3.1.2’ [81], 118 participants per major dementia subtype (i.e. AD and VaD) need to be recruited to ensure a 95% confidence interval with 80% power.

### Data analysis

We will use descriptive statistics for the demographic features of the cohort. The prevalence and intensity of pain and the causes of pain will be determined in the overall group of people with dementia. In addition, the frequency of pain subtypes per dementia subtype and dementia stage will be analyzed for group differences using Chi square tests.

For the secondary outcome analyses, descriptive statistics and multivariable linear regression analyses will be used. Multivariable linear regression will be used to evaluate the relationships between neuropsychiatric symptoms and pain, quality of life and pain, and cognitive functioning and pain, in dementia subtypes.

Differences in autonomic responses per dementia subtype, measured by the VU-AMS, as well as the effect of feedback on the presence of pain will be analyzed using paired t-tests.

## Discussion

The aim of the study is to investigate the prevalence and intensity of pain in the major dementia subtypes and for different dementia stages, as well as the relationship between the different types of pain and the major dementia subtypes. Primary outcome is the presence and intensity of pain in dementia subtypes. Secondary outcomes are oral health, autonomic responses to pain stimuli, vital sensitivity, cognitive functioning, neuropsychiatric symptoms, and quality of life*.* Additionally, an evaluation of effectiveness of feedback on the presence of pain will be carried out, and the possibility of using autonomic responses for assessing pain in patients with dementia will be explored.

In this study, a unique multidisciplinary approach is used, in which a neuropsychologist, dentist, and elderly care medicine trainee all examine the same group of participants. All participants will have a cognitive assessment, an oral examination and physical assessment. Results will be gathered and a differential diagnosis, including a treatment advice, if desired, will be provided to the attending physician.

We assume that the prevalence of pain will be different for the four most common dementia subtypes, and will be the highest in VaD and we expect to see more neuropathic pain in VaD, in which it is more likely to see neuropathic pain due to white matter lesions [12,82].

This study will provide data about the prevalence and intensity of orofacial pain and oral health problems in persons with dementia. Although there is an increase in attention for oral health in people with dementia, studies on oral health in people with dementia are scarce. Furthermore, available data indicate that oral health in people with dementia is poorer compared to cognitive intact people [83].

The chosen design seems suitable for our purposes. We will include patients in all different stages of the disease by recruiting at the outpatient memory clinic and in nursing homes, and we will also consider all possible causes of pain that are most likely to be diagnosed due to our multidisciplinary approach. The chosen measurement instruments are all commonly used in the field of elderly care medicine and geriatrics, except for the MOBID-2,OPS-NVI and the VU-AMS.

Observational pain assessment tools have limited validity and could lead to misinterpretation of pain behavior. For example, a grimace might be interpreted as a smile, or a positive score on an observational pain tool might be attributed to pain instead of some other cause of distress [20]. However, there is currently no better method of assessing pain in a population with severe dementia beyond the proposed approach in this study. We acknowledge that this may lead to an overdetection of pain in this study. As we use several observational instruments this enables to determine correlations between the instruments. This will give insight in the probability of overdetection and also enhance knowledge about the concurrent validity of the instruments.

For that reason we planned to investigate the contribution of autonomic responses to pain stimuli and QST as more objective measurement methods in the assessment of pain in people with dementia. The reliability of QST in people with dementia has already been demonstrated [21], although the use of QST in epidemiologic studies on pain in dementia is scarce.

The success of this study in nursing home residents depends highly upon support of the nursing staff. Participating in this research may be perceived as time-consuming by the nursing staff. Therefore, we decided to support the nurses by deploying research assistants. The research assistants will collect data on behavior, quality of life, participation, and ADL by interviewing the contact nurse to ensure that participation is as little time-consuming as possible. These research assistants will be trained in administering the instruments correctly and consistently.

In conclusion, this study will help to enhance our knowledge regarding the prevalence of different types of pain in different dementia subtypes i.e. AD, VaD, FTD and DLB. This study also aims to contribute to a better understanding of oral health problems in people with dementia and the relationships between pain and cognitive symptoms, neuropsychiatric symptoms, and quality of life in people with various dementia subtypes and in different stages of the disease.

## Abbreviations

AD: Alzheimer’s Disease
ADL: Activities of Daily Living
ADRDA: Alzheimer’s Disease and Related Disorders Association
AIREN: Association Internationale pour la Recherche et l’Enseignement en Neurosciences
AME: Active Mandibular Examination
BPI: Brief Pain Inventory
COGA: Centrum voor Ouderengeneeskunde Amsterdam
CT: Computed Tomography
DMFT: Decayed Missing Filled Teeth
DLB: Dementia with Lewy Bodies
DPSI: Dutch Periodontal Screening Index
DQoL: Dementia Quality of Life instrument
DSM: Diagnostic and Statistical Manual of Mental DisordersdZ: Thorax impedance
ECG: Electrocardiogram
FPS-R: Faces Pain Scale Revised
FTD: Frontotemporal Dementia
GDS: Global Deterioration Scale
IBI: Inter Beat Interval
MMSE: Mini Mental State Examination
MOBID-2: Mobilization Observation Behaviour Intensity Dementia 2 Pain Scale
MRI: Magnetic Resonance Imaging
NINCDS: National Institute of Neurological and Communicative Disorders and Stroke
NINDS: National Institute of Neurological Disorders and Stroke
NPI-Q: Neuropsychiatric Inventory Questionnaire
NPI-NH: Neuropsychiatric Inventory Nursing Home
NRS: Numeric Rating Scale
OPS-NVI: Orofacial Pain Scale for Non Verbal Individuals
OU: Occlusal Units
PEP: Pre Ejection Period
QST: Quantitative Sensory Testing
RISE: Revised Index for Social Engagement
RSA: Respiratory Sinus Arrhythmia
SCL: Skin Conductance Level
VaD: Vascular dementia
VAS: Visual Analogue Scale
VDS: Verbal Descriptor Scale
VU-AMS: Vrije Universiteit Ambulatory Monitoring System
